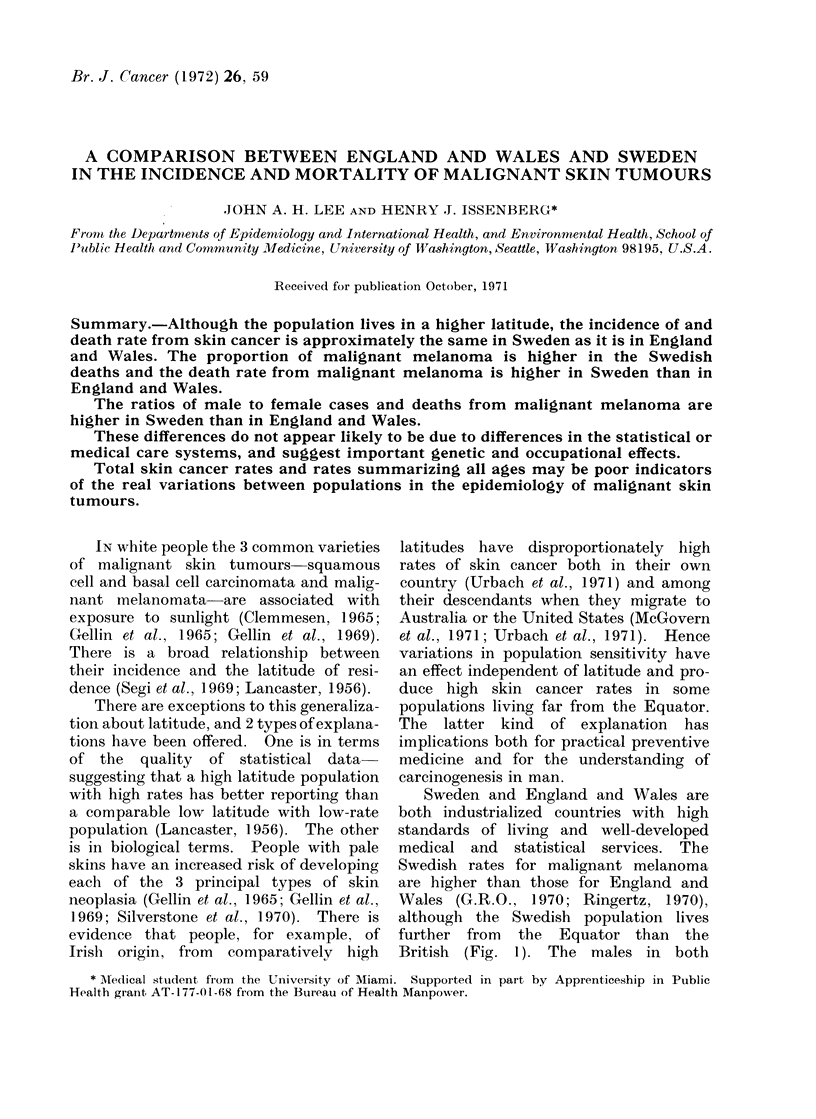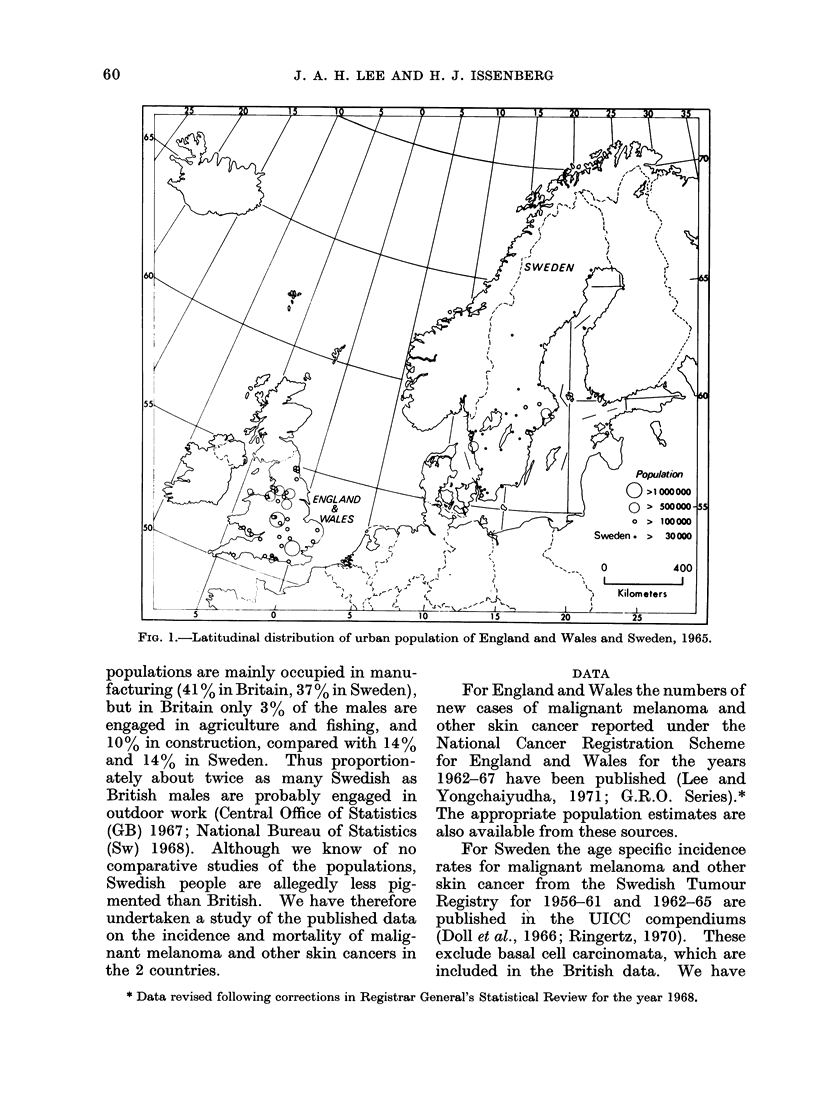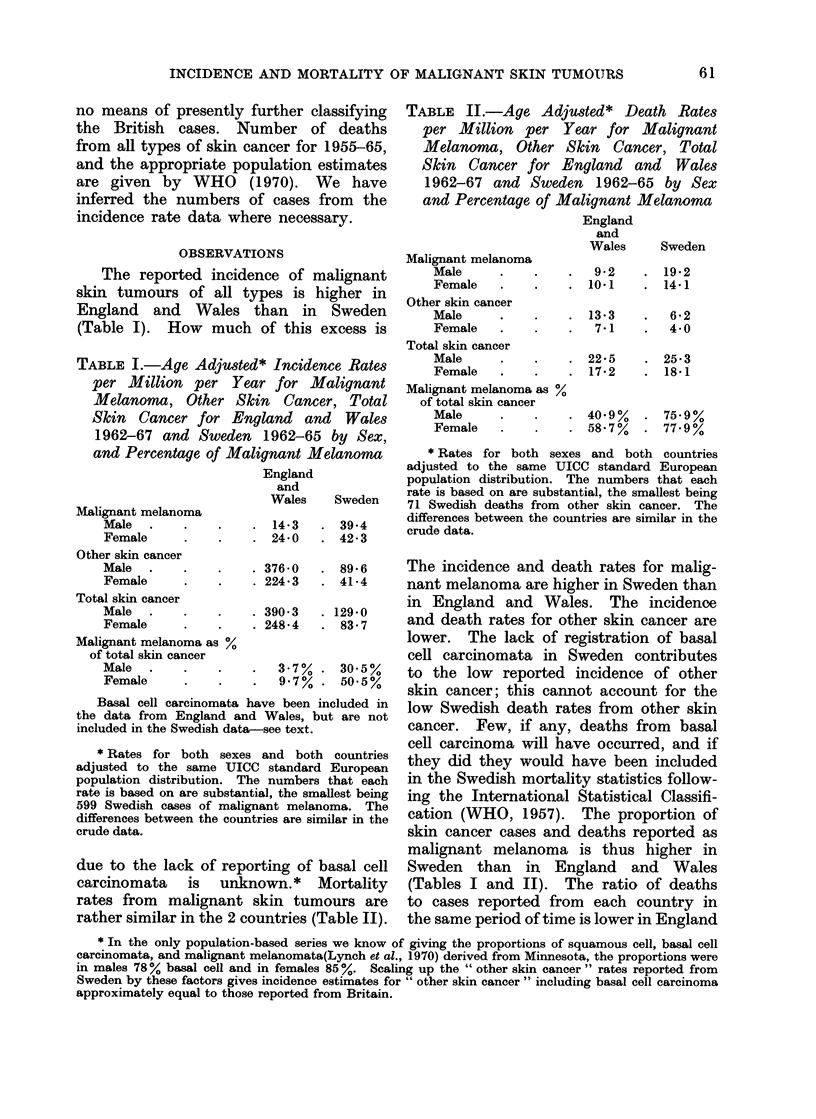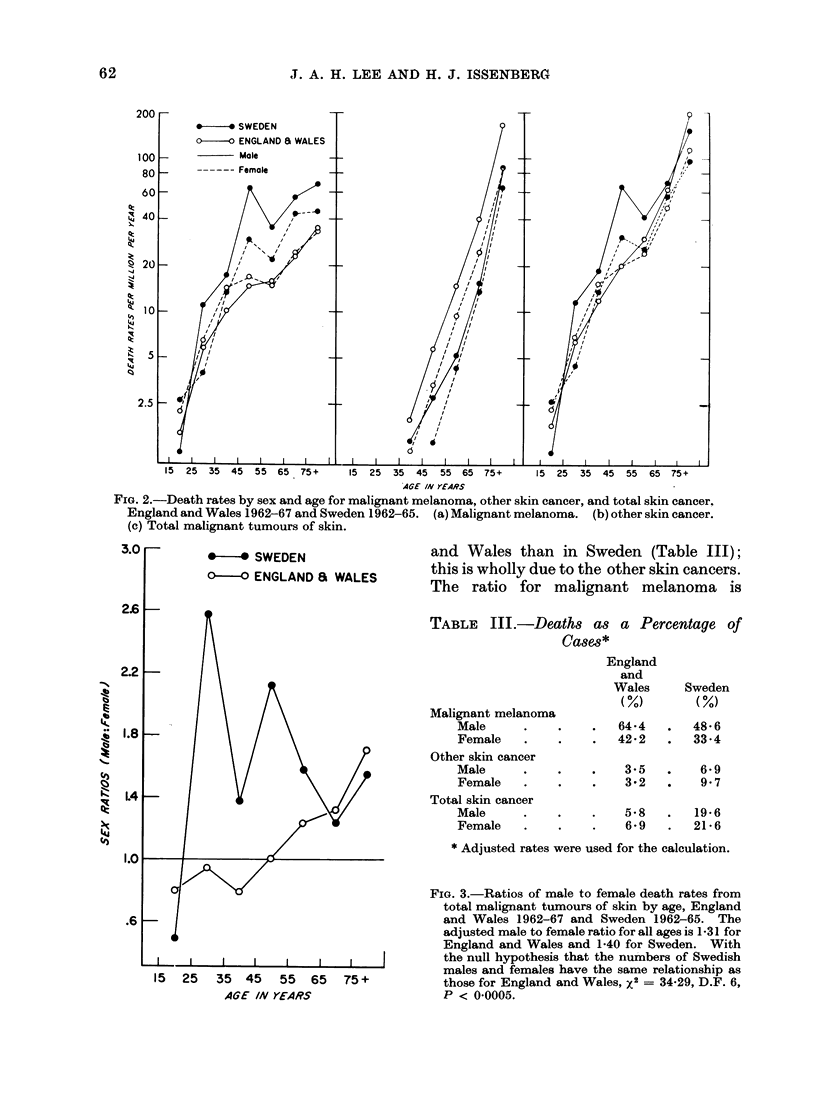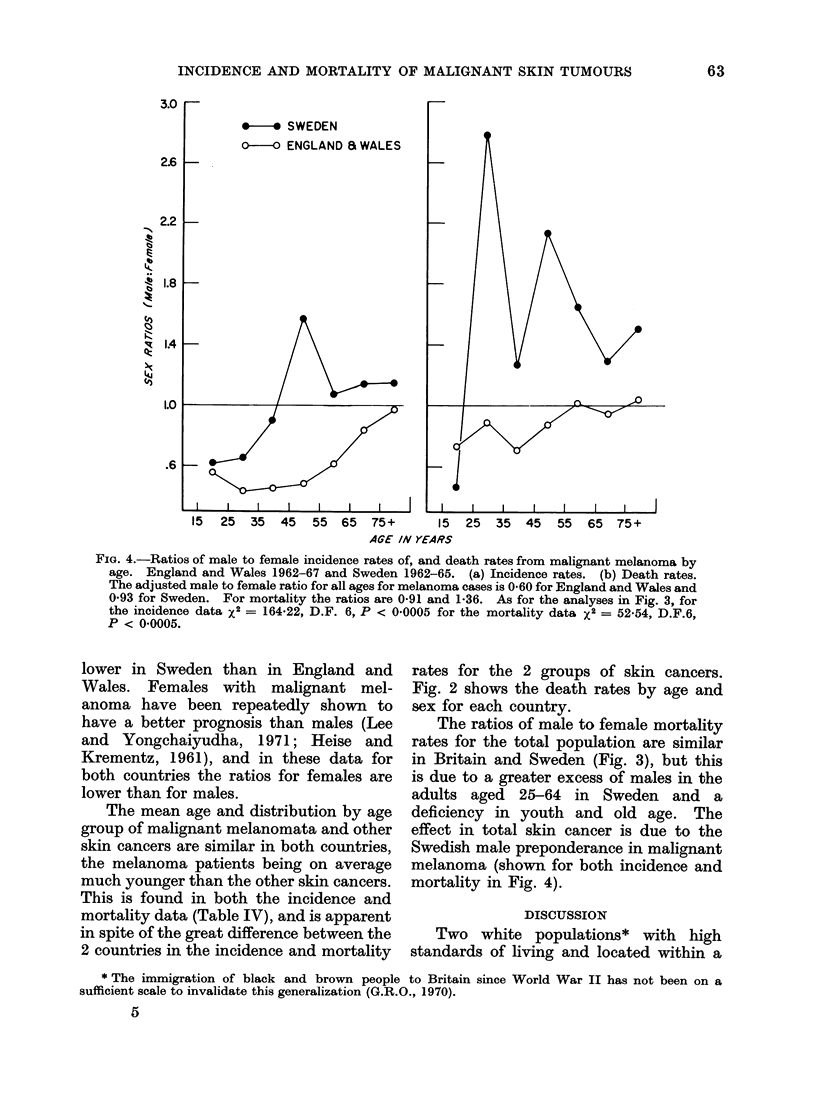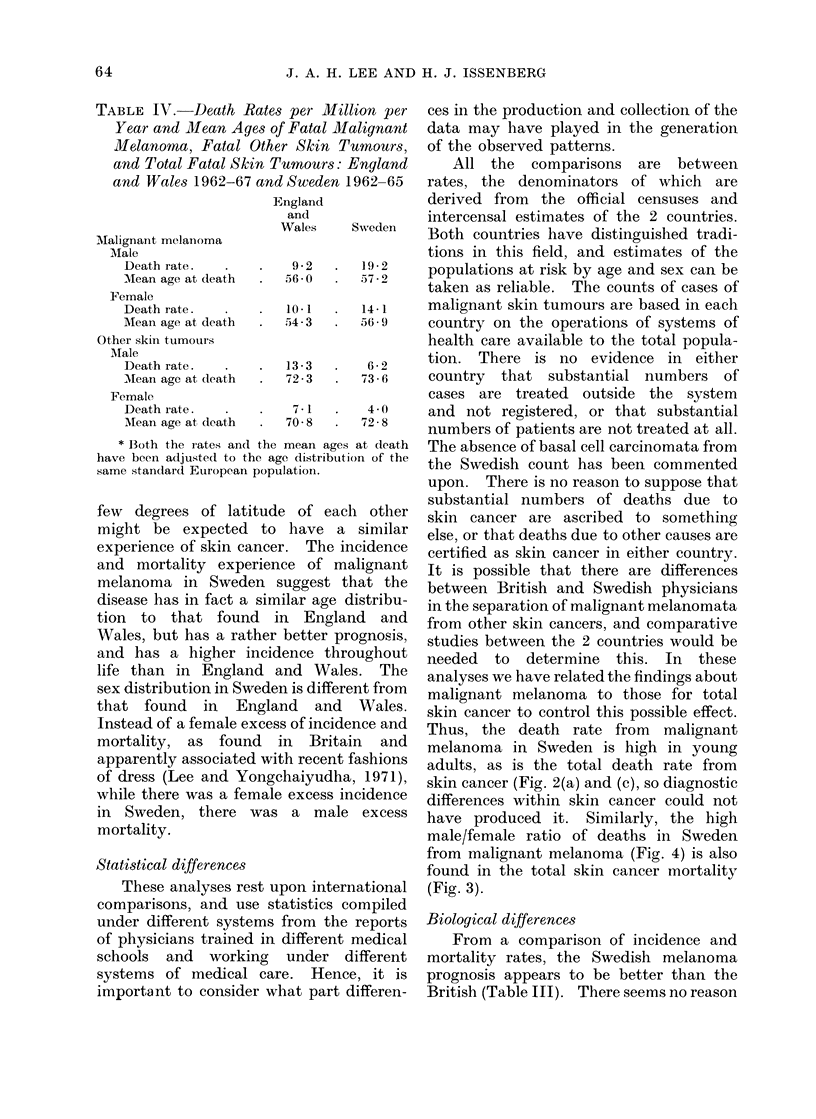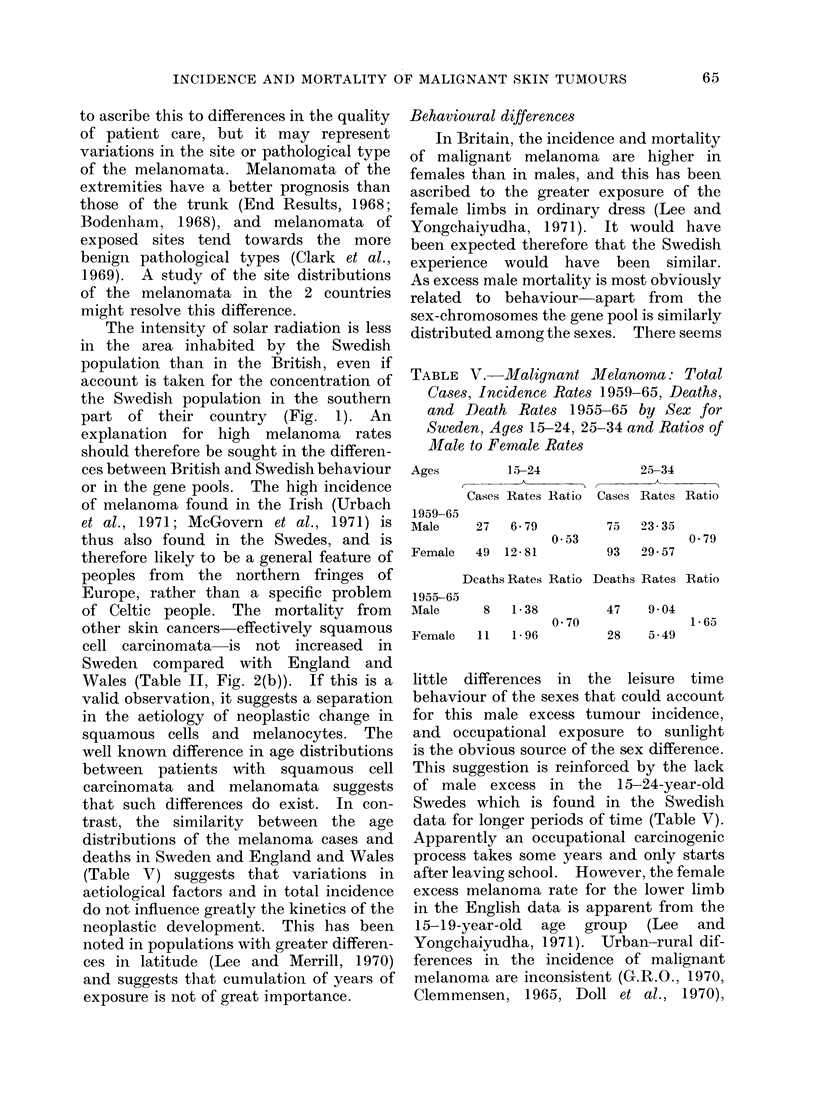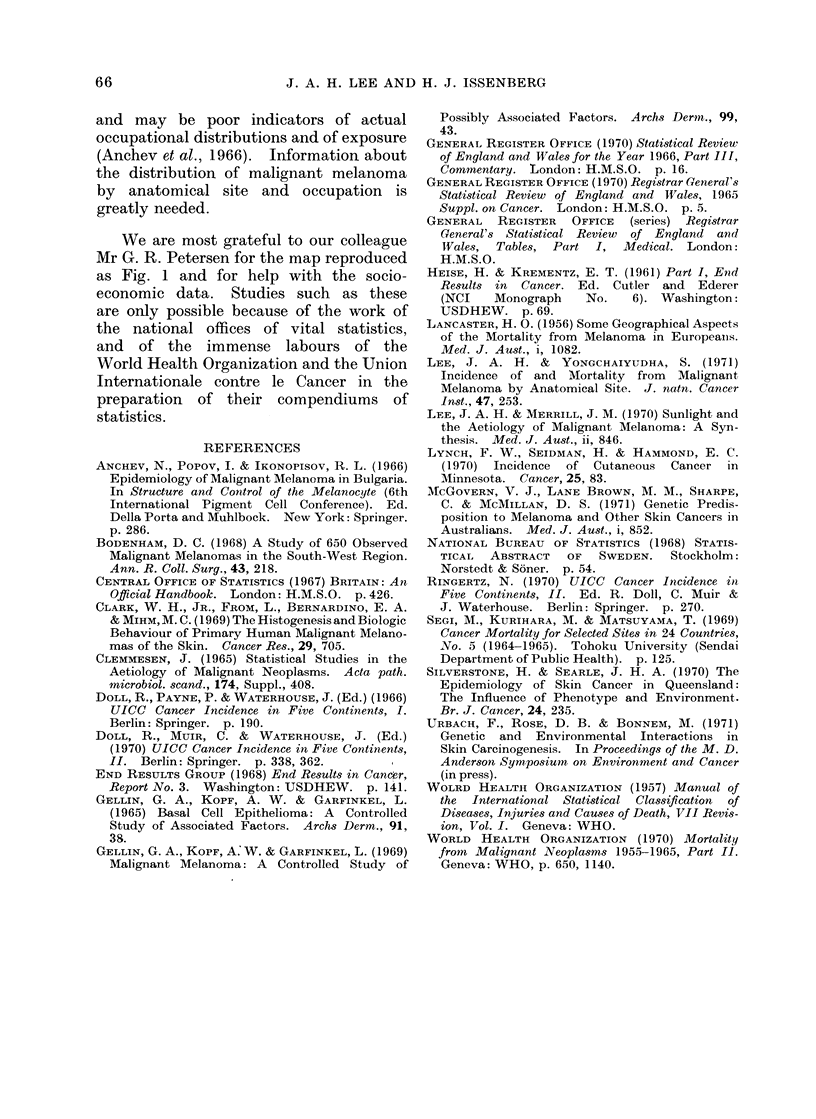# A Comparison Between England and Wales and Sweden in the Incidence and Mortality of Malignant Skin Tumours

**DOI:** 10.1038/bjc.1972.10

**Published:** 1972-02

**Authors:** John A. H. Lee, Henry J. Issenberg

## Abstract

Although the population lives in a higher latitude, the incidence of and death rate from skin cancer is approximately the same in Sweden as it is in England and Wales. The proportion of malignant melanoma is higher in the Swedish deaths and the death rate from malignant melanoma is higher in Sweden than in England and Wales.

The ratios of male to female cases and deaths from malignant melanoma are higher in Sweden than in England and Wales.

These differences do not appear likely to be due to differences in the statistical or medical care systems, and suggest important genetic and occupational effects.

Total skin cancer rates and rates summarizing all ages may be poor indicators of the real variations between populations in the epidemiology of malignant skin tumours.


*Br. J. Cancer* (1972) **26**, 59

### A COMPARISON BETWEEN ENGLAND AND WALES AND SWEDEN IN THE INCIDENCE AND MORTALITY OF MALIGNANT SKIN TUMOURS

JOHN A. H. LEE AND HENRY J. ISSENBERG*

*From the Departments of Epidemiology and International Health, and Environmental Health, School of Public Health and Community Medicine, University of Washington, Seattle, Washington* 98195, *U.S.A.*

Received for publication October, 1971

**Summary.—Although the population lives in a higher latitude, the incidence of and death rate from skin cancer is approximately the same in Sweden as it is in England and Wales. The proportion of malignant melanoma is higher in the Swedish deaths and the death rate from malignant melanoma is higher in Sweden than in England and Wales.**

**The ratios of male to female cases and deaths from malignant melanoma are higher in Sweden than in England and Wales.**

**These differences do not appear likely to be due to differences in the statistical or medical care systems, and suggest important genetic and occupational effects.**

**Total skin cancer rates and rates summarizing all ages may be poor indicators of the real variations between populations in the epidemiology of malignant skin tumours.**

IN white people the 3 common varieties of malignant skin tumours—squamous cell and basal cell carcinomata and malignant melanomata—are associated with exposure to sunlight (Clemmesen, 1965; Gellin *et al.*, 1965; Gellin *et al.*, 1969). There is a broad relationship between their incidence and the latitude of residence (Segi *et al.*, 1969; Lancaster, 1956).

There are exceptions to this generalization about latitude, and 2 types of explanations have been offered. One is in terms of the quality of statistical data—suggesting that a high latitude population with high rates has better reporting than a comparable low latitude with low-rate population (Lancaster, 1956). The other is in biological terms. People with pale skins have an increased risk of developing each of the 3 principal types of skin neoplasia (Gellin *et al.*, 1965; Gellin *et al.*, 1969; Silverstone *et al.*, 1970). There is evidence that people, for example, of Irish origin, from comparatively high latitudes have disproportionately high rates of skin cancer both in their own country (Urbach *et al.*, 1971) and among their descendants when they migrate to Australia or the United States (McGovern *et al.*, 1971; Urbach *et al.*, 1971). Hence variations in population sensitivity have an effect independent of latitude and produce high skin cancer rates in some populations living far from the Equator. The latter kind of explanation has implications both for practical preventive medicine and for the understanding of carcinogenesis in man.

Sweden and England and Wales are both industrialized countries with high standards of living and well-developed medical and statistical services. The Swedish rates for malignant melanoma are higher than those for England and Wales (G.R.O., 1970; Ringertz, 1970), although the Swedish population lives further from the Equator than the British (Fig. 1). The males in both

* Medical student from the University of Miami. Supported in part by Apprenticeship in Public Health grant AT-177-01-68 from the Bureau of Health Manpower.

FIG. 1.—Latitudinal distribution of urban population of England and Wales and Sweden, 1965.

populations are mainly occupied in manufacturing (41% in Britain, 37% in Sweden), but in Britain only 3% of the males are engaged in agriculture and fishing, and 10% in construction, compared with 14% and 14% in Sweden. Thus proportionately about twice as many Swedish as British males are probably engaged in outdoor work (Central Office of Statistics (GB) 1967; National Bureau of Statistics (Sw) 1968). Although we know of no comparative studies of the populations, Swedish people are allegedly less pigmented than British. We have therefore undertaken a study of the published data on the incidence and mortality of malignant melanoma and other skin cancers in the 2 countries.

### DATA

For England and Wales the numbers of new cases of malignant melanoma and other skin cancer reported under the National Cancer Registration Scheme for England and Wales for the years 1962–67 have been published (Lee and Yongchaiyudha, 1971; G.R.O. Series).* The appropriate population estimates are also available from these sources.

For Sweden the age specific incidence rates for malignant melanoma and other skin cancer from the Swedish Tumour Registry for 1956–61 and 1962–65 are published in the UICC compendiums (Doll *et al.*, 1966; Ringertz, 1970). These exclude basal cell carcinomata, which are included in the British data. We have

* Data revised following corrections in Registrar General's Statistical Review for the year 1968.

no means of presently further classifying the British cases. Number of deaths from all types of skin cancer for 1955–65, and the appropriate population estimates are given by WHO (1970). We have inferred the numbers of cases from the incidence rate data where necessary.

### OBSERVATIONS

The reported incidence of malignant skin tumours of all types is higher in England and Wales than in Sweden (Table I). How much of this excess is due to the lack of reporting of basal cell carcinomata is unknown.* Mortality rates from malignant skin tumours are rather similar in the 2 countries (Table II).

TABLE I.—*Age Adjusted* Incidence Rates per Million per Year for Malignant Melanoma, Other Skin Cancer, Total Skin Cancer for England and Wales* 1962–67 *and Sweden* 1962–65 *by Sex, and Percentage of Malignant Melanoma*

| | England and Wales | Sweden |
|---|---|---|
| Malignant melanoma | | |
| Male | 14·3 | 39·4 |
| Female | 24·0 | 42·3 |
| Other skin cancer | | |
| Male | 376·0 | 89·6 |
| Female | 224·3 | 41·4 |
| Total skin cancer | | |
| Male | 390·3 | 129·0 |
| Female | 248·4 | 83·7 |
| Malignant melanoma as % of total skin cancer | | |
| Male | 3·7% | 30·5% |
| Female | 9·7% | 50·5% |

Basal cell carcinomata have been included in the data from England and Wales, but are not included in the Swedish data—see text.

* Rates for both sexes and both countries adjusted to the same UICC standard European population distribution. The numbers that each rate is based on are substantial, the smallest being 599 Swedish cases of malignant melanoma. The differences between the countries are similar in the crude data.

TABLE II.—*Age Adjusted* Death Rates per Million per Year for Malignant Melanoma, Other Skin Cancer, Total Skin Cancer for England and Wales* 1962–67 *and Sweden* 1962–65 *by Sex and Percentage of Malignant Melanoma*

| | England and Wales | Sweden |
|---|---|---|
| Malignant melanoma | | |
| Male | 9·2 | 19·2 |
| Female | 10·1 | 14·1 |
| Other skin cancer | | |
| Male | 13·3 | 6·2 |
| Female | 7·1 | 4·0 |
| Total skin cancer | | |
| Male | 22·5 | 25·3 |
| Female | 17·2 | 18·1 |
| Malignant melanoma as % of total skin cancer | | |
| Male | 40·9% | 75·9% |
| Female | 58·7% | 77·9% |

* Rates for both sexes and both countries adjusted to the same UICC standard European population distribution. The numbers that each rate is based on are substantial, the smallest being 71 Swedish deaths from other skin cancer. The differences between the countries are similar in the crude data.

The incidence and death rates for malignant melanoma are higher in Sweden than in England and Wales. The incidence and death rates for other skin cancer are lower. The lack of registration of basal cell carcinomata in Sweden contributes to the low reported incidence of other skin cancer; this cannot account for the low Swedish death rates from other skin cancer. Few, if any, deaths from basal cell carcinoma will have occurred, and if they did they would have been included in the Swedish mortality statistics following the International Statistical Classification (WHO, 1957). The proportion of skin cancer cases and deaths reported as malignant melanoma is thus higher in Sweden than in England and Wales (Tables I and II). The ratio of deaths to cases reported from each country in the same period of time is lower in England

* In the only population-based series we know of giving the proportions of squamous cell, basal cell carcinomata, and malignant melanomata(Lynch *et al.*, 1970) derived from Minnesota, the proportions were in males 78% basal cell and in females 85%. Scaling up the " other skin cancer " rates reported from Sweden by these factors gives incidence estimates for " other skin cancer " including basal cell carcinoma approximately equal to those reported from Britain.

FIG. 2.—Death rates by sex and age for malignant melanoma, other skin cancer, and total skin cancer, England and Wales 1962–67 and Sweden 1962–65. (a) Malignant melanoma. (b) other skin cancer. (c) Total malignant tumours of skin.

and Wales than in Sweden (Table III); this is wholly due to the other skin cancers. The ratio for malignant melanoma is

TABLE III.—*Deaths as a Percentage of Cases**

| | England and Wales (%) | Sweden (%) |
|---|---|---|
| Malignant melanoma | | |
| Male . . . . | 64·4 | 48·6 |
| Female . . . . | 42·2 | 33·4 |
| Other skin cancer | | |
| Male . . . . | 3·5 | 6·9 |
| Female . . . . | 3·2 | 9·7 |
| Total skin cancer | | |
| Male . . . . | 5·8 | 19·6 |
| Female . . . . | 6·9 | 21·6 |

* Adjusted rates were used for the calculation.

FIG. 3.—Ratios of male to female death rates from total malignant tumours of skin by age, England and Wales 1962–67 and Sweden 1962–65. The adjusted male to female ratio for all ages is 1·31 for England and Wales and 1·40 for Sweden. With the null hypothesis that the numbers of Swedish males and females have the same relationship as those for England and Wales, $\chi^2 = 34{\cdot}29$, D.F. 6, $P < 0{\cdot}0005$.

FIG. 4.—Ratios of male to female incidence rates of, and death rates from malignant melanoma by age. England and Wales 1962–67 and Sweden 1962–65. (a) Incidence rates. (b) Death rates. The adjusted male to female ratio for all ages for melanoma cases is 0·60 for England and Wales and 0·93 for Sweden. For mortality the ratios are 0·91 and 1·36. As for the analyses in Fig. 3, for the incidence data $\chi^2 = 164{\cdot}22$, D.F. 6, $P < 0{\cdot}0005$ for the mortality data $\chi^2 = 52{\cdot}54$, D.F.6, $P < 0{\cdot}0005$.

lower in Sweden than in England and Wales. Females with malignant melanoma have been repeatedly shown to have a better prognosis than males (Lee and Yongchaiyudha, 1971; Heise and Krementz, 1961), and in these data for both countries the ratios for females are lower than for males.

The mean age and distribution by age group of malignant melanomata and other skin cancers are similar in both countries, the melanoma patients being on average much younger than the other skin cancers. This is found in both the incidence and mortality data (Table IV), and is apparent in spite of the great difference between the 2 countries in the incidence and mortality rates for the 2 groups of skin cancers. Fig. 2 shows the death rates by age and sex for each country.

The ratios of male to female mortality rates for the total population are similar in Britain and Sweden (Fig. 3), but this is due to a greater excess of males in the adults aged 25–64 in Sweden and a deficiency in youth and old age. The effect in total skin cancer is due to the Swedish male preponderance in malignant melanoma (shown for both incidence and mortality in Fig. 4).

### DISCUSSION

Two white populations* with high standards of living and located within a

* The immigration of black and brown people to Britain since World War II has not been on a sufficient scale to invalidate this generalization (G.R.O., 1970).

TABLE IV.—*Death Rates per Million per Year and Mean Ages of Fatal Malignant Melanoma, Fatal Other Skin Tumours, and Total Fatal Skin Tumours: England and Wales* 1962–67 *and Sweden* 1962–65

| | England and Wales | Sweden |
|---|---|---|
| Malignant melanoma | | |
| Male | | |
| Death rate . . . | 9·2 . | 19·2 |
| Mean age at death . | 56·0 . | 57·2 |
| Female | | |
| Death rate . . . | 10·1 . | 14·1 |
| Mean age at death . | 54·3 . | 56·9 |
| Other skin tumours | | |
| Male | | |
| Death rate . . . | 13·3 . | 6·2 |
| Mean age at death . | 72·3 . | 73·6 |
| Female | | |
| Death rate . . . | 7·1 . | 4·0 |
| Mean age at death . | 70·8 . | 72·8 |

* Both the rates and the mean ages at death have been adjusted to the age distribution of the same standard European population.

few degrees of latitude of each other might be expected to have a similar experience of skin cancer. The incidence and mortality experience of malignant melanoma in Sweden suggest that the disease has in fact a similar age distribution to that found in England and Wales, but has a rather better prognosis, and has a higher incidence throughout life than in England and Wales. The sex distribution in Sweden is different from that found in England and Wales. Instead of a female excess of incidence and mortality, as found in Britain and apparently associated with recent fashions of dress (Lee and Yongchaiyudha, 1971), while there was a female excess incidence in Sweden, there was a male excess mortality.

*Statistical differences*

These analyses rest upon international comparisons, and use statistics compiled under different systems from the reports of physicians trained in different medical schools and working under different systems of medical care. Hence, it is important to consider what part differences in the production and collection of the data may have played in the generation of the observed patterns.

All the comparisons are between rates, the denominators of which are derived from the official censuses and intercensal estimates of the 2 countries. Both countries have distinguished traditions in this field, and estimates of the populations at risk by age and sex can be taken as reliable. The counts of cases of malignant skin tumours are based in each country on the operations of systems of health care available to the total population. There is no evidence in either country that substantial numbers of cases are treated outside the system and not registered, or that substantial numbers of patients are not treated at all. The absence of basal cell carcinomata from the Swedish count has been commented upon. There is no reason to suppose that substantial numbers of deaths due to skin cancer are ascribed to something else, or that deaths due to other causes are certified as skin cancer in either country. It is possible that there are differences between British and Swedish physicians in the separation of malignant melanomata from other skin cancers, and comparative studies between the 2 countries would be needed to determine this. In these analyses we have related the findings about malignant melanoma to those for total skin cancer to control this possible effect. Thus, the death rate from malignant melanoma in Sweden is high in young adults, as is the total death rate from skin cancer (Fig. 2(a) and (c), so diagnostic differences within skin cancer could not have produced it. Similarly, the high male/female ratio of deaths in Sweden from malignant melanoma (Fig. 4) is also found in the total skin cancer mortality (Fig. 3).

*Biological differences*

From a comparison of incidence and mortality rates, the Swedish melanoma prognosis appears to be better than the British (Table III). There seems no reason to ascribe this to differences in the quality of patient care, but it may represent variations in the site or pathological type of the melanomata. Melanomata of the extremities have a better prognosis than those of the trunk (End Results, 1968; Bodenham, 1968), and melanomata of exposed sites tend towards the more benign pathological types (Clark *et al.*, 1969). A study of the site distributions of the melanomata in the 2 countries might resolve this difference.

The intensity of solar radiation is less in the area inhabited by the Swedish population than in the British, even if account is taken for the concentration of the Swedish population in the southern part of their country (Fig. 1). An explanation for high melanoma rates should therefore be sought in the differences between British and Swedish behaviour or in the gene pools. The high incidence of melanoma found in the Irish (Urbach *et al.*, 1971; McGovern *et al.*, 1971) is thus also found in the Swedes, and is therefore likely to be a general feature of peoples from the northern fringes of Europe, rather than a specific problem of Celtic people. The mortality from other skin cancers—effectively squamous cell carcinomata—is not increased in Sweden compared with England and Wales (Table II, Fig. 2(b)). If this is a valid observation, it suggests a separation in the aetiology of neoplastic change in squamous cells and melanocytes. The well known difference in age distributions between patients with squamous cell carcinomata and melanomata suggests that such differences do exist. In contrast, the similarity between the age distributions of the melanoma cases and deaths in Sweden and England and Wales (Table V) suggests that variations in aetiological factors and in total incidence do not influence greatly the kinetics of the neoplastic development. This has been noted in populations with greater differences in latitude (Lee and Merrill, 1970) and suggests that cumulation of years of exposure is not of great importance.

*Behavioural differences*

In Britain, the incidence and mortality of malignant melanoma are higher in females than in males, and this has been ascribed to the greater exposure of the female limbs in ordinary dress (Lee and Yongchaiyudha, 1971). It would have been expected therefore that the Swedish experience would have been similar. As excess male mortality is most obviously related to behaviour—apart from the sex-chromosomes the gene pool is similarly distributed among the sexes. There seems

TABLE V.—*Malignant Melanoma: Total Cases, Incidence Rates* 1959–65, *Deaths, and Death Rates* 1955–65 *by Sex for Sweden, Ages* 15–24, 25–34 *and Ratios of Male to Female Rates*

| Ages | 15–24 | | | 25–34 | | |
|---|---|---|---|---|---|---|
| | Cases | Rates | Ratio | Cases | Rates | Ratio |
| 1959–65 | | | | | | |
| Male | 27 | 6·79 | 0·53 | 75 | 23·35 | 0·79 |
| Female | 49 | 12·81 | | 93 | 29·57 | |
| | Deaths | Rates | Ratio | Deaths | Rates | Ratio |
| 1955–65 | | | | | | |
| Male | 8 | 1·38 | 0·70 | 47 | 9·04 | 1·65 |
| Female | 11 | 1·96 | | 28 | 5·49 | |

little differences in the leisure time behaviour of the sexes that could account for this male excess tumour incidence, and occupational exposure to sunlight is the obvious source of the sex difference. This suggestion is reinforced by the lack of male excess in the 15–24-year-old Swedes which is found in the Swedish data for longer periods of time (Table V). Apparently an occupational carcinogenic process takes some years and only starts after leaving school. However, the female excess melanoma rate for the lower limb in the English data is apparent from the 15–19-year-old age group (Lee and Yongchaiyudha, 1971). Urban–rural differences in the incidence of malignant melanoma are inconsistent (G.R.O., 1970, Clemmensen, 1965, Doll *et al.*, 1970), and may be poor indicators of actual occupational distributions and of exposure (Anchev *et al.*, 1966). Information about the distribution of malignant melanoma by anatomical site and occupation is greatly needed.

We are most grateful to our colleague Mr G. R. Petersen for the map reproduced as Fig. 1 and for help with the socio-economic data. Studies such as these are only possible because of the work of the national offices of vital statistics, and of the immense labours of the World Health Organization and the Union Internationale contre le Cancer in the preparation of their compendiums of statistics.

### REFERENCES

ANCHEV, N., POPOV, I. & IKONOPISOV, R. L. (1966) Epidemiology of Malignant Melanoma in Bulgaria. In *Structure and Control of the Melanocyte* (6th International Pigment Cell Conference). Ed. Della Porta and Muhlbock. New York: Springer. p. 286.

BODENHAM, D. C. (1968) A Study of 650 Observed Malignant Melanomas in the South-West Region. *Ann. R. Coll. Surg.*, **43**, 218.

CENTRAL OFFICE OF STATISTICS (1967) BRITAIN: *An Official Handbook.* London: H.M.S.O. p. 426.

CLARK, W. H., JR., FROM, L., BERNARDINO, E. A. & MIHM, M. C. (1969) The Histogenesis and Biologic Behaviour of Primary Human Malignant Melanomas of the Skin. *Cancer Res.*, **29**, 705.

CLEMMESEN, J. (1965) Statistical Studies in the Aetiology of Malignant Neoplasms. *Acta path. microbiol. scand.*, **174**, Suppl., 408.

DOLL, R., PAYNE, P. & WATERHOUSE, J. (Ed.) (1966) *UICC Cancer Incidence in Five Continents, I.* Berlin: Springer. p. 190.

DOLL, R., MUIR, C. & WATERHOUSE, J. (Ed.) (1970) *UICC Cancer Incidence in Five Continents, II.* Berlin: Springer. p. 338, 362.

END RESULTS GROUP (1968) *End Results in Cancer, Report No.* 3. Washington: USDHEW. p. 141.

GELLIN, G. A., KOPF, A. W. & GARFINKEL, L. (1965) Basal Cell Epithelioma: A Controlled Study of Associated Factors. *Archs Derm.*, **91**, 38.

GELLIN, G. A., KOPF, A. W. & GARFINKEL, L. (1969) Malignant Melanoma: A Controlled Study of Possibly Associated Factors. *Archs Derm.*, **99**, 43.

GENERAL REGISTER OFFICE (1970) *Statistical Review of England and Wales for the Year* 1966, *Part III, Commentary.* London: H.M.S.O. p. 16.

GENERAL REGISTER OFFICE (1970) *Registrar General's Statistical Review of England and Wales,* 1965 *Suppl. on Cancer.* London: H.M.S.O. p. 5.

GENERAL REGISTER OFFICE (series) *Registrar General's Statistical Review of England and Wales, Tables, Part I, Medical.* London: H.M.S.O.

HEISE, H. & KREMENTZ, E. T. (1961) *Part I, End Results in Cancer.* Ed. Cutler and Ederer (NCI Monograph No. 6). Washington: USDHEW. p. 69.

LANCASTER, H. O. (1956) Some Geographical Aspects of the Mortality from Melanoma in Europeans. *Med. J. Aust.*, i, 1082.

LEE, J. A. H. & YONGCHAIYUDHA, S. (1971) Incidence of and Mortality from Malignant Melanoma by Anatomical Site. *J. natn. Cancer Inst.*, **47**, 253.

LEE, J. A. H. & MERRILL, J. M. (1970) Sunlight and the Aetiology of Malignant Melanoma: A Synthesis. *Med. J. Aust.*, ii, 846.

LYNCH, F. W., SEIDMAN, H. & HAMMOND, E. C. (1970) Incidence of Cutaneous Cancer in Minnesota. *Cancer,* **25**, 83.

MCGOVERN, V. J., LANE BROWN, M. M., SHARPE, C. & MCMILLAN, D. S. (1971) Genetic Predisposition to Melanoma and Other Skin Cancers in Australians. *Med. J. Aust.*, i, 852.

NATIONAL BUREAU OF STATISTICS (1968) STATISTICAL ABSTRACT OF SWEDEN. Stockholm: Norstedt & Söner. p. 54.

RINGERTZ, N. (1970) *UICC Cancer Incidence in Five Continents, II.* Ed. R. Doll, C. Muir & J. Waterhouse. Berlin: Springer. p. 270.

SEGI, M., KURIHARA, M. & MATSUYAMA, T. (1969) *Cancer Mortality for Selected Sites in 24 Countries, No.* 5 (1964–1965). Tohoku University (Sendai Department of Public Health). p. 125.

SILVERSTONE, H. & SEARLE, J. H. A. (1970) The Epidemiology of Skin Cancer in Queensland: The Influence of Phenotype and Environment. *Br. J. Cancer,* **24**, 235.

URBACH, F., ROSE, D. B. & BONNEM, M. (1971) Genetic and Environmental Interactions in Skin Carcinogenesis. In *Proceedings of the M. D. Anderson Symposium on Environment and Cancer* (in press).

WOLRD HEALTH ORGANIZATION (1957) *Manual of the International Statistical Classification of Diseases, Injuries and Causes of Death, VII Revision, Vol. I.* Geneva: WHO.

WORLD HEALTH ORGANIZATION (1970) *Mortality from Malignant Neoplasms* 1955–1965, *Part II.* Geneva: WHO, p. 650, 1140.